# Abstract of an Experimental Investigation into the Functions of the Eighth Pair of Nerves

**Published:** 1838-01

**Authors:** John Reid


### ABSTRACT OF AN EXPERIMENTAL INVESTIGATION INTO THE FUNCTIONS OF THE EIGHTH PAIR OF NERVES. BY JOHN REID, M.D.

[A SHORT account of the enquiry the chief results of which we are about to present to our readers, was laid before the medical section of the British Association at its late meeting in Liverpool; and we are happy to be now enabled to give such an abstract of it as may convey an idea of its value. We owe this gratification to the kindness of the author, who has transmitted to us a copy of the original paper, which appears, we believe, in full, in the January number of our able contemporary, the Edinburgh Med. and Surg. Journal. The difficulty of attaining satisfactory conclusions on this intricate subject is allowed by all, and by none more completely than by Dr. R. himself. He has, however, brought to his task qualifications of no ordinary kind: the most accurate anatomical knowledge and operative skill, the most careful and candid observation of phenomena, the most judicious discrimination regarding their causes, and the most philosophical spirit in reasoning upon them. That our praise is exaggerated, no one, we venture to predict, will affirm after reading the original paper. It is incumbent upon all experimentalists, who arrive at results different from those usually obtained, to point out, as far as they have the means, the causes of the discrepancy; and this the acuteness of Dr. R. has enabled him to do in a great proportion of the instances in which he questions the accuracy of the conclusions of other physiologists. We feel the greatest confidence in the accuracy of his inferences, not only on the grounds already mentioned, but because he never carries them a step further than his facts absolutely warrant; always reserving for future investigation any points which he does not regard as fully determined. The number of the experiments, also, and the correspondence of their phenomena *under similar circumstances*, are a strong guarantee for their accuracy; whilst the care taken to ascertain the precise nature of the operation performed by *subsequent minute dissection*, often disclosed unexpected causes for apparent diversity in the results. It is only necessary for us further to state, that the names of Dr. Alison and Dr. Sharpey frequently present themselves as additional testimonies to the accuracy of Dr. Reid's statements.

The following brief account of the results of each set of experiments is principally derived from the recapitulations made by Dr. R. himself in the original memoir. We reserve for future notice some of the more interesting questions regarding the associated actions in which these nerves are concerned. We are not the less willing to lay these results of Dr. Reid's investigations before our readers, because they tend to invalidate some opinions formerly expressed by us: we can have only one object, the attainment of truth.]

GLOSSO-PHARYNGEAL NERVE. From twenty-seven experiments upon this nerve, the following conclusions are drawn:

1. That it is a nerve of *common sensation*, as indicated by the unequivocal expression of pain by the animal when the nerve is pricked, pinched, or cut.

2. That the extensive muscular movements of the throat and lower parts of the face, which follow the mechanical or chemical irritation either of the pharyngeal branches themselves or of the trunk in which these are united, depend, not upon any influence extending downwards along the branches of the nerve to the muscles moved, but to a reflex action, transmitted through the central organs of the nervous system. This is clearly proved by the following statement: "It was repeatedly observed that, when the nerve was cut across, irritation of the lower end, or that in connexion with the muscles, was followed by no movement; while, on the other hand, irritation of the cranial end was followed by as powerful convulsive twitches as when the nerve was entire." It is also shown that this irritation is conveyed by the pharyngeal branches only; that the pinching of the lingual branch gives rise to no muscular movements; whilst these effects are strongly produced by irritation of small twigs of the pharyngeal division.

3. That irritation of this nerve immediately after death is not followed by any muscular movements, when sufficient care is taken to insulate it from the pharyngeal branch of the par vagum; whilst, at the same time, irritation of the divided extremity of the latter nerve will produce these movements.

4. That the pharyngeal branches of the glosso-pharyngeal possess endowments connected with the peculiar sensations of the mucous membranes upon which they are distributed, though it is not yet possible to specify in what these consist; but that this cannot be the sole nerve upon which all these sensations depend, since the perfect division of the trunk on both sides does not interfere with the perfect performance of the function of deglutition.

5. That it may participate with other nerves in the performance of the function of taste, but that it certainly is not the special nerve of that sense, since, after the perfect section of both trunks, the sense of taste is sufficiently acute to enable the animal readily to recognize bitter substances; and that the sensation of thirst does not entirely depend upon it.

Pneumogastric Nerve. *Pharyngeal Branches.* From the previous and other experiments, it also appears:

1. That these branches are the motor nerves of the constrictors of the pharynx, the stylo-pharyngeus, and the muscles of the soft palate, and are called upon to act in obedience to the stimulus conveyed by the glosso-pharyngeal and other sensory nerves.

2. That they have little or nothing to do with sensation, no decided indications of suffering being produced by irritating them.

3. That section of this nerve greatly impairs the power of deglutition; the animal retaining, however, the means of forcing small morsels through the pharynx by the action of the muscles of the tongue and neck.

*Œsophageal Branches.* The following appears satisfactorily ascertained regarding the function of these branches:

That the continuity of these nerves with the spinal cord is necessary for the action of the œsophageal muscular fibres, which do not act by direct stimulation, as Haller supposed; and that a clear case of *reflex action without sensation* is thus established.

On the *Gastric branches*, Dr. Reid's observations are not yet concluded, but we believe that they will probably lead to a similar result.

*Laryngeal branches.* From numerous experiments on these nerves, Dr. Reid draws the following interesting and novel conclusions:

1. That the superior laryngeal furnishes one muscle only with *motor* filaments, viz. the crico-thyroid.

2. That it furnishes all, or nearly all, the *sensitive* filaments of the larynx, and also some of those distributed upon the *mucous surface* of the larynx.

3. That the inferior laryngeal, or recurrent, furnishes the sensitive filaments to the upper part of the trachea, a few to the mucous surface of the *pharynx,* and still fewer to the mucous surface of the *larynx.*

4. That, when any irritation is applied to the mucous membrane of the larynx in a healthy state, this does not excite the contraction of the muscles which move the arytenoid cartilages, by acting directly upon these through the mucous membrane; but that this contraction takes place by a reflex action, in the performance of which the superior laryngeal is the excitor, and the inferior laryngeal is the motor nerve.

*Cardiac branches.* It seems probable, from Dr. Reid's experiments, that these branches form one of the means by which the heart's action is influenced by the state of the cerebro-spinal nervous system; since sudden destruction of the brain did not produce so great an effect upon the motion when these nerves had been divided as in animals in which they were entire. It would seem, however, that the sympathetic is the medium of communication for impression upon the spinal cord.

*Pulmonary branches.* Dr. Reid fully confirms the statements of other experimentalists, that respiratory movements continue after the section of the pneumogastric nerves; though he believes that they ordinarily convey the stimulus to the medulla oblongata by which these movements are excited. He has fully proved that the continued movements cannot be of a voluntary character as Dr. Hall supposes; and seems inclined to refer them to the action of the sympathetic, in which view we think that he is borne out by comparative anatomy, since the two nerves are much more closely united in many of the lower animals than in man. Dr. R. doubts whether the mucous membrane of the trachea and bronchi is sensitive to the extent stated by Brâchet; and has given a very interesting account of the changes produced in the lungs by section of these nerves, to which we are prevented by want of space from referring more particularly.

Spinal Accessory Nerve. Dr. Reid denies that this can be regarded as specially a respiratory nerve, and states that its *external* branch seems to resemble in its functions the filaments of the cervical plexus, with which it anastomoses freely; whilst the *internal* branch assists in moving the muscles of the pharynx. Its other endowments Dr. R. intends to make the subject of future experiments.

